# Maternal Healthcare Financing: Gujarat's Chiranjeevi Scheme and Its Beneficiaries

**DOI:** 10.3329/jhpn.v27i2.3367

**Published:** 2009-04

**Authors:** Ramesh Bhat, Dileep V. Mavalankar, Prabal V. Singh, Neelu Singh

**Affiliations:** ^1^ Indian Institute of Management, Vastrapur, Ahmedabad 380 015, India; ^2^ Maternal Health Division, Department of Health, Government of Gujarat, India

**Keywords:** Chiranjeevi scheme, Delivery, Emergency obstetric care, Maternal mortality, Obstetric care, Private-public relationship, India

## Abstract

Maternal mortality is an important public-health issue in India, specifically in Gujarat. Contributing factors are the Government's inability to operationalize the First Referral Units and to provide an adequate level of skilled birth attendants, especially to the poor. In response, the Gujarat state has developed a unique public-private partnership called the Chiranjeevi Scheme. This scheme focuses on institutional delivery, specifically emergency obstetric care for the poor. The objective of the study was to explore the targeting of the scheme, its coverage, and socioeconomic profile of the beneficiaries and to assess financial protection offered by the scheme, if any, in Dahod, one of the initial pilot districts of Gujarat. A household-level survey of beneficiaries (n=262) and non-users (n=394) indicated that the scheme is well-targeted to the poor but many poor people do not use the services. The beneficiaries saved more than Rs 3,000 (US$ 75) in delivery-related expenses and were generally satisfied with the scheme. The study provided insights on how to improve the scheme further. Such a financing scheme could be replicated in other states and countries to address the cost barrier, especially in areas where high numbers of private specialists are available.

## INTRODUCTION

It is estimated that about 1.2 million children in the Gujarat state are born each year, including both institutional and domiciliary deliveries. Given that the maternal mortality rate for the state is estimated at 172 per 100,000 livebirths (1), an estimated 2,064 of these mothers die from maternal causes. The primary reason for these maternal deaths is that the majority of deliveries are not attended by skilled persons, women do not have access to emergency obstetric care (EmOC), and there is little postnatal follow-up. It is argued that most of these maternal deaths are avoidable with adequate interventions, such as skilled birth attendant (SBA), referral services, and access to EmOC (2-5). Families below the poverty-line (BPL) are the most vulnerable since they face significant risk due to their poor socioeconomic status and limited access to healthcare services (6).

The Chiranjeevi Scheme, implemented by the Government of Gujarat, aims at encouraging the BPL families to access institutional delivery at a private hospital. This is done by providing financial protection to these families and covering their out-of-pocket costs incurred on travel to reach the healthcare facility. The scheme also provides financial support to the accompanying person for loss of wages. The scheme uses several mechanisms to target the BPL family, the main mechanism being the BPL card. This card is issued to families earning less than a particular level of income and certain asset ownership criteria. The BPL card helps identify this group of population for provision of various benefits and to target the benefits.

The Chiranjeevi Scheme was launched as a one-year pilot project in December 2005 in five backward districts: Banaskantha, Dahod, Kutch, Panchmahals, and Sabarkantha (7). The scheme has now been extended to the entire state. When the scheme was initiated, the pilot districts were selected based on remoteness and included regions with the highest rate of infant mortality. The private medical practitioners (mainly obstetricians) in these districts were empanelled in the scheme to provide delivery-care services to BPL women. These care providers are reimbursed on a fixed rate for deliveries carried out by them (8). Details of the financial package for the Chiranjeevi Scheme are given in Table 1. The objective of the study was to explore the targeting of the scheme, its coverage, and socioeconomic profile of the beneficiaries and to assess financial protection offered by the scheme, if any, in Dahod, one of the initial pilot districts of Gujarat.

**Table 1.** Details of the financial package for the Chiranjeevi scheme (7)

Services, procedure, and benefits	Cases per 100 deliveries	Cost (Rs) per procedure	Total payment (Rs) per 100 deliveries
Normal delivery	85	800	68,000
Complicated cases			
Eclampsia/forceps/vacuum/breech	3	1,000	3,000
Septicaemia	2	3,000	6,000
Blood transfusion	3	1,000	3,000
Caesarean section	7	5,000	35,000
Pre-delivery visit (ANC)	100	100	10,000
Other costs			
Investigation	100	50	5,000
Sonography	30	150	4,500
NICU support	10	1,000	10,000
Food	100	100	10,000
*Dai* (accompanying person)	100	50	5,000
Transport (cash payment to mother)	100	200	20,000
Total	100		1,79,500

ANC=Antenatal care; NICU=Neonatal Intensive Care Unit

## MATERIALS AND METHODS

The Chiranjeevi Scheme was implemented on a pilot basis in five districts, including Dahod, starting in December 2005.

**Fig. 1.**
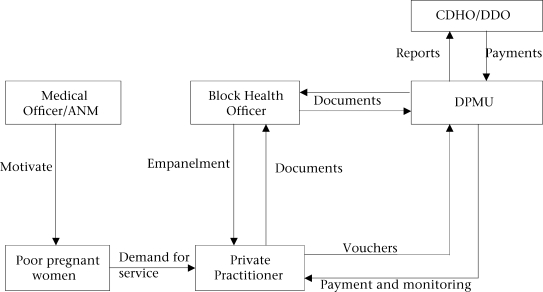
Flow diagram of implementation of the Chiranjeevi Scheme in Dahod

The scheme involved creating a panel of private care providers who would accept referrals by the families covered under the scheme (9). Identification and empanelment of the private obstetricians were done by the Block Health Officer (BHO). After the private practitioner agrees to join the scheme, a Memorandum of Understanding is signed between him/her and the district health authorities. The District Project Management Unit (DPMU) for the Reproductive and Child Health (RCH) programme handles all documentations for the scheme. The DPMU is also responsible for reporting progress of the scheme to the State Health Directorate and for making payments to the empanelled obstetricians through the District Development Officer (DDO) and Chief District Health Officer (CDHO). A flow diagram of implementation of the scheme is described in Figure 1.

ANM=Auxiliary Nurse Midwife; CDHO=Chief District Health Officer; DDO=District Development Officer; DPMU=District Project Management Unit

The medical officers and the Auxiliary Nurse Midwife (ANM) of the respective Subcentres undertake the responsibility of motivating the community (BPL families) to take the benefit from the scheme. The ANM visits the communities and registers a pregnant woman for receiving antenatal care (ANC); at that time, she motivates women to avail of the Chiranjeevi Scheme. She encourages the pregnant woman to visit an empanelled obstetrician. Every month, the empanelled providers present their filled-in vouchers for deliveries conducted and claim their reimbursement. The entire document for the reimbursement is submitted at DPMU; the DPMU initiates the process of payment. Payment is made after approval of CDHO and DDO.

### Selection of sample

All five pilot districts were put in three groups based on their geographical proximity to each other. Group 1 was selected as it had the largest number of deliveries among the three groups. Within Group 1, Dahod was selected as it had the higher average number of deliveries per care provider (Table 2). Group 1 is also more backward, has more tribal population, and geographically in the east of the state. Group 2 districts are in the north of the state, are somewhat backwards, and has some tribal population in one of them. Group 3 has only one district of Kutch, which is geographically large, thinly populated, poor, and deserted on north-west side of the state, bordering Pakistan.

**Table 2.** District data on obstericians available, enrolled in the scheme and deliveries conducted in 5 pilot districts of Chiranjeevi scheme, 2006 (10)

District	Geographical group	Total specialist (obstetricians) in the district	Specialist empanelled under Chiranjeevi Scheme	Total no. of deliveries conducted under Chiranjeevi Scheme (till November 2006)	Average no. of deliveries per care provider
Panchmahals	Group 1	29	29	10,450	360
Dahod		18	15	6,750	450
Banaskantha	Group 2	50	58	5,945	103
Sabarkantha		73	10	4,584	458
Kutch	Group 3	47	21	3,912	186
Total		217	133	31,641	238

### District profile

The population of Dahod was 1,751,000 in 2006 when the study was implemented. The BPL population of the district was about 23% (11). The total number of deliveries in Dahod district was about 41,500 during 2006 assuming a crude birth rate of 23.7 (12) and even distribution of births over all economic groups. Using this as the basis, the estimated number of BPL deliveries was about 9,545 per annum. During 2006, 7,735 deliveries were conducted under the Chiranjeevi Scheme. Hence, the number of deliveries under Chiranjeevi accounts for about 81% of all estimated deliveries by BPL women. This is based on the assumption that the BPL cards were issued only to the targeted population. The percentage of deliveries under Chiranjeevi Scheme was about 18.6 of all deliveries in the district.

The total number of deliveries conducted in the district between December 2005 (when the scheme started) and March 2007 was 9,854, of which 7,584 were normal, 391 by caesarean section (lower segment caesarean section), and 1,879 complicated. Month-wise data are presented in Table 3. On average, about 615 deliveries were conducted under the Chiranjeevi scheme in Dahod district per month.

**Table 3.** Chiranjeevi deliveries in Dahod (1 December 2005–28 March 2007)

Nature of deliveries				
Month	Normal	Caesarean section (LSCS)	Complicated	Total
December 2005	83	20	31	134
January 2006	158	16	53	227
February 2006	356	20	117	493
March 2006	423	23	167	613
April 2006	325	2	30	357
May 2006	402	19	65	486
June 2006	592	21	95	708
July 2006	623	26	146	795
August 2006	529	26	98	653
September 2006	575	31	154	760
October 2006	592	24	130	746
November 2006	745	35	184	964
December 2006	764	55	114	933
January 2007	693	45	183	921
February 2007	348	20	164	532
up to 28 March 2007	376	8	148	532
Grand total	7,584	391	1,879	9,854

LSCS=Lower segment caesarean section

**Fig. 2.**
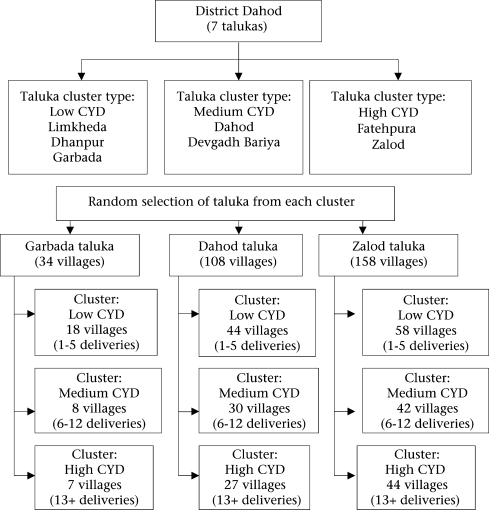
Sampling process within Dahod district

Within Dahod district, a multi-stage hierarchical cluster-sampling procedure was adopted to select households for data collection (Fig. 2). Data for the number of deliveries under the Chiranjeevi Scheme conducted in all seven talukas (subdistrict) of the district from January to December 2006 were obtained from the DHO of the district. In the first stage of sampling, talukas of the district were classified as low, moderate, and high based on the number of deliveries under the Chiranjeevi Scheme. Three clusters of talukas were, thus, formed, and one taluka from each cluster was randomly picked. In the second stage, villages falling under these three talukas were further classified into three clusters (low, moderate, and high) based on the number of deliveries conducted under the Chiranjeevi Scheme. Sample households were randomly selected in proportion to deliveries from the three clusters. The list of the households was obtained from the ANM/FHW of the respective areas. Listing of pregnant women with ANM/FHW is quite comprehensive and is the most exhaustive list available for sampling purpose. Two types of samples were selected: (a) beneficiaries of the scheme defined as mothers who delivered in private obstetrician clinics and benefited from the scheme (Chiranjeevi beneficiaries—CB), and (b) non-beneficiary mothers of the Chiranjeevi Scheme (NCM) defined as poor women (BPL) who did not benefit from the scheme. The sample size for the Chiranjeevi Scheme beneficiaries was fixed at around 250, given the available resources. As an estimated 20% of the BPL deliveries are taking place outside the Chiranjeevi Scheme, it was decided to oversample the NCM group. The final NCM sample was 394.

CYD: Chiranjeevi delivery

### Questionnaire development

Initially, a pilot questionnaire was prepared which was later revised based on the field-testing. The questionnaire was divided into 10 sections, of which the first five, covering background characteristics, were the same for both CB and NCM. Section 6 (obstetric history of recent delivery in the Chiranjeevi Scheme), 7 (details of neonatal care of the recent Chiranjeevi delivers), and 8 (knowledge and practice of Chiranjeevi Scheme) were administered only to CB clients. For CB clients, information was collected for both index and previous deliveries whereas, in the case of NCM, only information on their index (most recent) delivery was collected.

All the field investigators had prior experience in conducting field interviews in rural settings in the district-level household surveys. They were trained for two days, of which the first day was on the structure of the questionnaire and the second day was for conducting mock interviews. In all, there were four investigators conducting about 14-16 interviews per day. The whole exercise of field survey took about two months. There was one field editor and one supervisor whose jobs were to validate the questionnaire in the field and check for logical inconsistencies.

## RESULTS

A summary of the comparisons of CB and NCM on background variables is provided in Table 4.

**Table 4.** Comparison of beneficiaries and non-beneficiaries of the Chiranjeevi Scheme

Indicator	Chiranjeevi beneficiary (CB) (n=262)	Non-Chiranjeevi mothers (NCM) (n=394)	*t* value for comparisons between the two groups
Mean age (years) at marriage	17.98	18.12	-1.039
Mean age (average in years) at the time of previous delivery	22.5	-	-
Average age (years) at index delivery	24.53	25.05	-1.443∗∗
Average annual income (Rs)	7440.46	7365.93	0.672
Annual income below Rs 12,000 (%)	93.89	96.45	
Earning members (average)	2.08	2.39	-2.915∗
Dwelling (*kuchha* or hut) (%)	68	68	1.115
Average land-holding (hectares)	1.071	1.055	0.579
Land-holding (up to 5 hectares) (%)	94.74	95.56	
Education (% without formal education)	65.73	66.68	-0.263
Antenatal care complication (%)	48.80	52.70	-5.65∗
Total number of deliveries	2.53	2.84	-2.251∗
Place of last delivery (public institutions) (%)	2.67	1.78	-
Place of last delivery (private institutions) (%)	97.32	77.15	
Delivery in the home (%)	0.38	21.07	
Delivery conducted by private qualified obstetrictian/gynaecologist (%)	39.30	32.23	-0.629
% of livebirths	98.85	98.47	-0.333
% of livebirths, still living	96.56	98.47	-0.29
% of normal deliveries	94.6	97.2	-0.291
% taken postnatal care	28.24	30.71	-0.694
Complications during the postnatal period (%)	10.30	26.14	-0.538

∗Significant at 5%

∗∗Significant at 10%

### Sociodemographic profile

**Age at marriage and parity:** The mean age at marriage for the CB and non-Chiranjeevi mothers was 17.98 years and 18.12 years respectively. The average number of children born to CB was 2.53 compared to 2.84 for the NCM group. This difference is significant at 5%.

**Education:** The percentages of respondents without any kind of formal education were 66 for CB and 67 for NCMs. As per the Census 2001, this figure for the women in Gujarat state stands at 41.4%. Both CB and NCMs had a similarly low level of education, far lower than the average of Gujarat.

### Targeting based on income

In our study, 94% of the CB were having annual income below Rs 12,000 (US$ 300). This shows that the large majority of the beneficiaries are poor, and only 6% of the beneficiaries are non-poor. This also shows a high level of targeting of the scheme to the poor. As the NCM were also selected from the category of the poor, our study showed that 96% of them had annual income below Rs 12,000.

### Out-of-pocket expenditure and financial protection

#### Expenditure on delivery

Chiranjeevi beneficiaries are not supposed to bear any expense relating to delivery, as the Government pays all the costs of obstetrician directly, and they are to pay the women for transport and funds to the accompanying person. To assess this aspect of the scheme, the survey collected information on expenditure incurred by the CB and the NCM group. These expenses were further analyzed in terms of expenditure incurred on self and child's medicines, transportation costs, and other out-of-pocket expenditure.

To understand the financial protection/benefit received by the CB, we have compared the expenditure by the CB in the previous delivery and the expenditure by the NCM in their present (most recent) deliveries for a normal or complicated delivery at a private facility. This is to make them comparable as deliveries under Chiranjeevi exclusively take place in private facilities (Table 5). The CB, an average, incurred an expenditure of Rs 3,070 on their previous institutional delivery; this is before the scheme started. During their index delivery, 80% of the Chiranjeevi clients indicated spending, on average, Rs 655 on buying medicines for the neonate and self: Rs 296 for self and Rs 358 for the neonate. The out-of-pocket additional expenditure for CB on medicines is about 36% of the cost of the Chiranjeevi package, i.e. Rs 1,795 paid to the care provider per case basis. The Chiranjeevi beneficiaries are getting Rs 200 for transportation from the service provider as per the scheme benefit. They reported spending, on average, Rs 272, thus incurring an additional out-of-pocket expenditure of Rs 72. After including the transportation cost, the total out-of-pocket expenditure was Rs 727 for CBs which is on top of the government expenditure of Rs 1,795 (Table 5).

**Table 5.** Expenditure incurred on delivery of CB and NCM for delivering in the private facility

Item of expenditure and average cost (Rs)	CB index	CB previous		NCM current (private deliveries only)†	
	Package	Normal	Complicated	Normal	Complicated
Consultation charges	1,795∗	1,057	6,267	1,102	9,375
Medicine costs (out-of-pocket)	655	336	1,278	331	2,138
Bed charges	(included in 1,795)	26	144	47	50
Transport-cost (out-of-pocket)	72 (272-200)	257	329	276	369
Other charges	0	110	0	35	111
Total out-of-pocket cost	727	2,135	8,373	2,319	13,524
Overall average cost	2,522 (1,795+727)	3,070		4,000	

∗Total package paid to the doctor by the Government

†NCM who delivered at home are excluded; CB=Chiranjeevi beneficiaries; NCM=Non-beneficiary mothers of Chiranjeevi Scheme

The NCM spent, on average, Rs 2,319 for a normal delivery. This amount includes consultancy and procedure fees of Rs 1,102 paid to the doctor, transportation cost of Rs 276, and medicine cost of Rs 331. In most recent complicated deliveries, the NCM spent Rs 13,524 (detailed break up is given in Table 5). The weighted average for normal and complicated delivery cost for NCM came to be Rs 4,000.

Table 5 shows that the amount saved by the CB by availing of the benefit of the scheme is estimated at Rs 3,273 (Rs 4,000 [total expenditure of NCM] minus Rs 727, additional expenditure of CB). As per the design of the Chiranjeevi package, there is a provision of Rs 100 for providing diet to clients during hospitalization. Our survey showed that only 8.8% of the Chiranjeevi clients were provided diet during their stay in the healthcare facility.

### Quality of care

#### Antenatal care

Of all the clients who took benefit of the Chiranjeevi Scheme, 96% had gone for ANC services. The average number of ANC visits made by the clients was 2.84. The ANMs provided ANC services to 61% of the clients—16% were provided services by private doctors, and 2% were provided services by government doctors. About 17% of the clients received ANC services from more than one source.

Almost 49% of the CB reported antenatal problems compared to 53% in the NCM group. Of those who had antenatal problems, 71% went to private and 16% to a government hospital. Only 2% of the clients received intervention for ANC complications at home while 3% did not seek intervention for ANC complications faced by them.

**Transportation, distance travelled, and time taken:** The Chiranjeevi clients used rickshaw (most commonly), jeep, and *chhakdo* (an indigenous mechanized mode of transportation) to reach the healthcare facility for delivery. All modes of transportation are motorized, and most are private. About 93% of the respondents used a private mode of transportation. No government ambulance was used for transportation. On average, the CB travelled 13.8 (range 1-72) km and took 44 minutes (range 10 minutes–9 hours) to reach the facility.

### Delivery services

Although all deliveries under the Chiranjeevi Scheme are supposed to be in the hospital of private empanelled doctors, only one delivery (0.38%) of the CB group was conducted in the home, and 2.7% of deliveries were conducted in a government institution. All other (97%) deliveries were conducted in a private health facility. As far as the NCM is concerned, 21% of deliveries were conducted in the home, 1.8% in a government institution, and about 77% in private institutions.

About 94% of deliveries conducted in the CB group were normal; in the case of the NCM group, this figure was 97%. There was some indication that more complicated deliveries were going to the Chiranjeevi Scheme.

### Service providers

The percentage of deliveries conducted by private doctors was 41 in the case of CB and 32 in the case of NCM deliveries. Nurses and other trained attendants at a private facility conducted deliveries in 48% of the CB cases. This percentage for the NCM group was 43. The key difference here lies in the deliveries conducted by trained and untrained attendants. In the case of CB, TBAs conducted 1% of deliveries whereas, in the case of NCM, TBAs conducted 20% of deliveries.

### Postnatal care

Data showed that about 28% of the CB went for postnatal care (PNC); the corresponding percentage for the NCM group was around 31%.

### Awareness about the Chiranjeevi Scheme

**Awareness generation:** Of those clients who took advantage of the scheme CCB group), 55% were informed about the Chiranjeevi Scheme by the ANM/FHW and 17% by the *Anganwadi* workers (AWW—community-level nutrition worker). Public-health facilities, including the Subcentre, Primary Health Centres (PHCs), Community Health Centres (CHCs), and the district hospital, were the sources of information for only 6% of clients. Friends/neighbours were the sources of information for 4% in the CB group. Printed material and pamphlets were the sources of information for only 1% of clients. TBAs informed only 1% of the beneficiaries about the scheme. Others, such as *panchayats* (governing bodies at the village level) members, *balwadi* teachers, doctors, and nurses provided information to 6% of the CB. The questionnaire did not seek this information from the NCMs.

**BPL cards:** All clients who took the benefit of the scheme were aware about the requirement of the BPL card/certificate for availing of services. All beneficiaries mentioned that they possessed a BPL card. Also, 98% of the beneficiaries reported that their BPL cards were inspected at the healthcare facility prior to availing of services under the scheme.

### Satisfaction of clients

About 89% of the CB and 87% of the NCMs were satisfied with services provided at the health facilities. The main reason for satisfaction of 66% of the Chiranjeevi beneficiaries was ‘good quality of services' while the main reason for satisfaction of about 18% of the CB was ‘good facilities.' The reasons for satisfaction of 7% and 5% of the CB were, respectively, for ‘good behaviour of staff' and ‘prompt services'.

Eighty-six percent of the Chiranjeevi beneficiaries reported that the doctor was available when they reached the facility; only 1% reported that the doctor was not available; and the remaining beneficiaries stated they did not remember. In the case of the NCM, the doctor was available for 86% of the cases when they reached the facility.

Sixty percent of the CB group found that medicines were always available, and 30% reported that medicines were available most of the time. In both CB and NCM cases, 87% of the respondents expressed that the staff was courteous.

The respondents who used the Chiranjeevi Scheme were asked to provide suggestions for improving the scheme. The availability of medicines was identified as one important factor to improve services. Around a quarter of the clients suggested providing medicines to the beneficiaries under the Chiranjeevi Scheme to improve it. About 12% of the clients suggested payment of increased compensation for transportation (Rs 200). Around 5% of the beneficiaries also reported that the transportation expenses are not being provided to them.

Only about 4% of the beneficiaries reported that the nurses at the health facility asked for money, and this should be addressed. Another suggestion was to improve proximity of the community to the healthcare facility.

## DISCUSSION

### Targeting

Possession of the BPL cards is the criteria for selection of beneficiaries for the Chiranjeevi Scheme, and all Chiranjeevi beneficiaries had a BPL card. Ninety-four of the CB have an annual income below Rs 12,000 which comes to Rs 32.90 a day, less than the World Bank poverty-level of a dollar a day (equivalent to Rs 40). This suggests that the scheme achieved its objective of targeting the poor. Leakages or benefits to the non-poor were limited (about 6%). The NCMs were also equally poor but did not avail of the benefits of the scheme. In this study, the non-beneficiaries were also selected from the population eligible for the scheme, which are the people living below the poverty-line.

### Financial protection

Compared to the average expenditure of Rs 4,000 incurred by the NCM in their index delivery, the CB spent only Rs 727, thereby saving Rs 3,273. This saving is accruing to the poor, thereby increasing their health equity substantially.

### Expenditure

Additional expenditure incurred by the Chiranjeevi clients on medicines for self and child was, on average, Rs 654. This may indicate that the package offered to the private doctors at Rs 1,795 may have to be re-evaluated and enhanced. Second, better monitoring is needed as to why the doctors ordered additional medicines.

### Postnatal care

PNC needs to be strengthened as only 30% used it. One reason for this may be that after delivery was conducted, the service provider considered their job done. The Chiranjeevi Scheme currently focuses only on delivery care. The empanelled practitioner is just reimbursed for the delivery he/she conducts. The package under the scheme does not include payment to the provider for delivering PNC, and hence, it is overlooked by them. Even the public-health system neglects PNC as indicated by data from national surveys. Clients are also unaware of the need for PNC. The public-health staff (ANM/FHW) is supposed to do a follow-up of all Chiranjeevi clients for PNC. Unfortunately, no systematic data were kept for PNC by district health office. If there is early postpartum haemorrhage (PPH) just after the birth, it may be treated by the Chiranjeevi doctor but late PPH (after going home) may be missed.

### Role of health workers

It can be inferred from the data that the ANM/FHW and the AWW were the most common source of providing information and building awareness about the Chiranjeevi Scheme among the beneficiaries. It should also be noted that all these health workers have acted as an essential ‘link' between the healthcare-delivery system and the beneficiaries. The health workers have not only made the services available to the beneficiaries but also guided them on how to access these services.

The decision for choosing the place of delivery for the majority of the Chiranjeevi clients has also been taken by the ANM. The health workers are either from the community itself or are well-known to the community, and therefore, the community places a lot of faith in them. The health workers can be developed as a more important link in the healthcare-delivery system even if actual services are provided by private doctors.

### Quality of care and satisfaction of clients

It is heartening to note that most clients of the Chiranjeevi Scheme and non-clients were quite satisfied with delivery-related services. They also reported positive behaviour from the service provider and the staff. This shows that the scheme is able to provide client-pleasing services at almost half of the cost of the regular private-sector charges. Further research should address the technical quality of care.

### Improved healthcare-seeking behaviour

This scheme encourages poor women to deliver in a healthcare facility; for many, it is likely that they have accessed health services at an institution for the first time. Given their high level of satisfaction, they are likely to use services in the future for themselves and their children as well as recommend to others.

### Summary and conclusion

The Chiranjeevi Scheme has provided financial protection against the cost of delivery and EmOC to the marginalized section of the population. In the study, it was seen that a Chiranjeevi client saves around Rs 3,273 (about US$ 82) in delivery compared to those who did not avail of the benefits of the scheme. However, the scheme is not 100% cost-free to the BPL families as they had to pay out-of-pocket expenses for medicines and transportation. The Government spends Rs 1,795 (US$ 45) for each delivery. Thus, by buying the services in bulk from private care providers, the Government is getting delivery services at a much lower price than the market rates being paid by non-beneficiaries (Rs 4,000 or US$ 100). The findings suggest that the scheme needs to be strengthened by improving some aspects, including more funds for medicines, transportation, etc. and offering at least two antenatal and two postnatal visits.

The monitoring of the scheme needs to be improved in several ways. The client should not be made to pay any extra money; the doctor should maintain proper records and follow standard evidence-based protocols. Deaths of mothers and children need to be systematically documented and analyzed. Referrals made by the private care providers should be analyzed to ensure that they are not doing so to avoid more expensive treatments. Future studies are required to assess the technical quality of care and mortality impact of the programme. Women not using the services yet should be motivated to take benefit of the scheme. The private care providers should collaborate with institutions to develop an appropriate costing framework to implement this scheme and develop an understanding of whether all components of the programme are incorporated adequately. Unfortunately, private care providers are often reluctant to share their cost information which is vital to develop adequate understanding of the pricing issues in such a scheme.

The findings of the study suggest that, given the demographic characteristics of this district and the economic profile of the clients using this scheme, wrong targeting is not a major issue. About 94% of the Chiranjeevi clients earn much less than a dollar a day. Further study is needed to see as to why many of the poor who should be covered are left out of the scheme.

The government health employees, such as ANMs/FHWs, have been found to be effective in building awareness and guiding clients to use private services. Their role in the process has been found to be quite important and needs to be strengthened.

Overall, the study has shown that the scheme reaches the poor and provides substantial benefits to them. Based on the learning from this scheme, the Government of India and other developing countries can think of replicating this scheme on larger scale. The Government of Gujarat should consider expanding the scheme and its coverage to people who are just above the poverty-line.

## ACKNOWLEDGEMENTS

The authors thank Dr. Amarjit Singh, Commissioner Health and the Government of Gujarat for encouraging them to undertake the study. The survey carried out for the purpose of this paper was supported by the funds provided by the Department for International Development (DFID), UK. Its contents, however, are solely the responsibility of the authors and do not represent the official views of DFID. They also thank ICDDR,B through which the DFID funds were made available. The authors acknowledge the support of the district health administration team of Dahod led by Chief District Health Officer Dr. (Mrs.) D.V. Rathore. They are also thankful to Mr. Kapil Dev Singh, District Programme Coordinator, Dahod, for extending support in conducting the study. The authors also thank Vardaan Consultants—Vadodara and Dr. Harshit Sinha and his team for helping the authors in collecting the primary data from Dahod district. They also thank IIM Director and administration for their support.
